# Physical Activity and Improvement of Glycemia in Prediabetes by Different Diagnostic Criteria

**DOI:** 10.1210/jc.2017-00990

**Published:** 2017-07-26

**Authors:** Kristine Færch, Daniel Rinse Witte, Eric John Brunner, Mika Kivimäki, Adam Tabák, Marit Eika Jørgensen, Ulf Ekelund, Dorte Vistisen

**Affiliations:** 1 Steno Diabetes Center Copenhagen, 2820 Gentofte, Denmark; 2 Department of Public Health, Aarhus University, 8000 Aarhus, Denmark; 3 Danish Diabetes Academy, 5000 Odense, Denmark; 4 Department of Epidemiology and Public Health, University College London, London WC1E 6BT, United Kingdom; 5 First Department of Medicine, Faculty of Medicine, Semmelweis University, 1083 Budapest, Hungary; 6 National Institute of Public Health, Southern Denmark University, 1353 Copenhagen, Denmark; 7 Department of Sport Medicine, Norwegian School of Sport Sciences, 0806 Oslo, Norway; 8 Norwegian Institute of Public Health, 0403 Oslo, Norway

## Abstract

**Context:**

The effects of physical activity (PA) on improvement of glycemia may differ between prediabetic individuals defined by oral glucose tolerance test vs glycated hemoglobin (HbA_1c_).

**Objective:**

We studied the association between PA and improvement of glycemia in individuals with prediabetes defined by glucose vs HbA_1c_ criteria.

**Design, Setting, and Participants:**

From the Whitehall II study, 957 participants with prediabetes defined by isolated impaired fasting glucose (i-IFG), isolated impaired glucose tolerance (i-IGT), or both and 457 with prediabetes defined by HbA_1c_ were included.

**Main Outcome Measures:**

The associations of PA with concomitant changes in glucose-related outcomes during 5 years of follow-up were analyzed. A recursive partitioning analysis was performed to study heterogeneity in the association between baseline PA and the probability of reversion to normoglycemia.

**Results:**

After 5 years of follow-up, 405 (42%) individuals with glucose-defined prediabetes reverted to normal glucose tolerance (NGT). A 5-year increase in moderate-to-vigorous-intensity PA was associated with improvements in insulin sensitivity and *β*-cell function, but PA was not generally associated with reversion to NGT. Only among women ≥50 years with i-IFG or i-IGT, higher amounts of PA were associated with higher probability of reversion to NGT. In HbA_1c_-defined prediabetes, only 20 individuals (4.4%) reverted to normoglycemia, and PA was not associated with improvement in glycemic markers.

**Conclusions:**

PA may be particularly important for reversion to normoglycemia among older women with i-IFG or i-IGT. Individuals with prediabetes identified by HbA_1c_ have a low probability of reversion to normoglycemia, and their changes in glycemia are not related to PA.

## Teaser

In a prospective cohort study, individuals with prediabetes identified by HbA_1c_ had a low probability of reversion to normoglycemia. Their changes in glycemia were not related to physical activity.

Intermediate hyperglycemia, also known as prediabetes, is associated with a high risk of developing type 2 diabetes and cardiovascular disease (1). Prediabetes can be defined by measuring fasting plasma glucose (FPG) and/or 2-hour plasma glucose (2hPG) concentration during an oral glucose tolerance test (OGTT) (2, 3). More recently, also glycated hemoglobin (HbA_1c_) has been adopted as a diagnostic tool to identify individuals with prediabetes (2).

Strong evidence suggests that lifestyle modification, including improvement in physical activity (PA), can effectively lower diabetes and cardiovascular risk in individuals with impaired glucose tolerance (IGT) (4–7). The evidence is less clear for individuals with isolated impaired fasting glycemia (i-IFG) (8, 9) or people classified by the HbA_1c_ criterion. Studies have also shown that low levels of PA are predominantly associated with metabolic defects related to IGT (systemic insulin resistance and 2-hour hyperglycemia) as compared with i-IFG (defective first-phase insulin secretion, decreased basal hepatic glucose uptake, and fasting hyperglycemia) (9–12). Furthermore, the Diabetes Prevention Program (DPP) showed that lifestyle intervention was more effective than metformin for 2hPG-defined diabetes, whereas metformin and lifestyle intervention had a similar impact on FPG concentrations (4, 13). Together these findings suggest that individuals with i-IFG may not have the same benefits on glucose regulation from increasing PA as those with IGT (14). Studies examining the effect of PA on markers of glucose regulation in individuals with HbA_1c_-defined prediabetes are lacking.

The ultimate goal of diabetes prevention efforts is to reduce the risk of future diabetes, cardiovascular disease, and premature death. Results from the DPP showed that prediabetic individuals who normalized their blood glucose levels during the trial had significantly lower diabetes and cardiovascular risk than those who maintained their prediabetes status during the study (15, 16). Accordingly, the ability to restore normal glucose regulation can be used as a marker of an individual’s future risk. We hypothesized that the effects of PA on improvement of glycemia are different in prediabetic individuals defined by OGTT compared with HbA_1c_ criteria. We also hypothesized that within the group of prediabetic individuals defined by the OGTT, those with i-IFG have a smaller effect of daily PA on improvement of glycemia than individuals with isolated impaired glucose tolerance (i-IGT) or IFG+IGT. Thus, the overall objective of this study was to examine heterogeneity in the association between PA and improvement in glycemia across different diagnostic methods and within the OGTT method. Specific aims were (1) to assess the strength of the association of 5-year changes in PA with concomitant changes in the levels of FPG, 2hPG, HbA_1c_, insulin sensitivity, and *β*-cell function in prediabetes defined by the glucose vs HbA_1c_ criteria, and (2) to examine potential heterogeneity in the association of baseline PA with reversion to normoglycemia in prediabetic subgroups and across age, sex, and obesity degree.

## Materials and Methods

### Study participants

Participants are from the Whitehall II study, an occupational cohort of 10,308 British civil servants (6896 men, 3412 women) initially recruited in 1985. The study population consists of the 6479 people participating in at least two consecutive phases (5-year observation windows) of the phases 5, 7, and/or 9 and without known diabetes at their first measurement. These phases are chosen because information on PA was not available before phase 5.

For the analysis of prediabetes by the OGTT criteria, we further excluded 6263 (34.0%) examinations for which the participants had been fasting for fewer than 8 hours and 1415 (7.7%) examinations without both fasting and 2-hour glucose measurements. Following this, 3348 participants remained with valid 5-year follow-up data, of which 957 (28.6%) had prediabetes at baseline according to the American Diabetes Association (ADA) glucose criteria (2) and were included in this study.

As HbA_1c_ was not measured at phase 5, the study population for analysis of prediabetes by the HbA_1c_ criteria is based on the 5601 people participating at both phases 7 and 9 and without known diabetes or HbA_1c_ ≥6.5% at phase 7. We further excluded 698 participants (12.5%) without HbA_1c_ measurement at both baseline and follow-up, leaving 4903 participants free of diabetes at baseline. Of these, 457 (9.3%) with prediabetes at baseline according to the ADA HbA_1c_ criteria (2) were included in this study.

### Measures of PA

A modified version of the previously validated Minnesota Leisure-Time Physical Activity Questionnaire was used to describe typical weekly PA [metabolic equivalent (MET) hours per week] (17). The questionnaire assessed both leisure-time and job-related activities, but with more focus on leisure-time PA. The questionnaire included 20 items on the amount of time spent in the following activities: walking, sports, gardening, housework, do-it-yourself activity, and other activities. For each item, the participants were requested to provide the total number of hours spent in that particular activity over the past 4 weeks. Subsequently, for each activity, a MET value was assigned by using a compendium of activity energy costs (18). One MET value reflects the metabolic cost during rest. The intensity of PA was classified using multiples of 1 MET; light-intensity physical activity (LPA) was defined as activities >1.5 METs and <3.0 METs (*e.g.*, dishwashing), and moderate-to-vigorous-intensity physical activity (MVPA) as activities ≥3.0 METs (*e.g.*, cycling or swimming). The total number of MET-hours per week spent in LPA and MVPA were calculated. Total physical activity (TPA) was defined as the sum of LPA and MVPA.

### Definition of prediabetes and measures of glycemia

At the clinical examinations at phases 5, 7, and 9, a standard 75-g OGTT was performed in the morning after ≥8 hours of fasting or in the afternoon after no more than a light breakfast eaten before 8:00 am (≥5 hours of fasting). Blood samples were drawn before and 2 hours after the glucose ingestion. Prediabetes was classified according to the ADA fasting and 2-hour OGTT glucose criteria after ≥8 hours of fasting (2). I-IFG was defined as FPG 5.6 to 6.9 mmol/L and 2hPG <7.8 mmol/L, i-IGT as FPG <5.6 and 2hPG 7.8 to 11.0 mmol/L, and combined IFG+IGT as FPG ≥5.6 and 2hPG ≥7.8 mmol/L. At phases 7 and 9, HbA_1c_ was measured and prediabetes was defined according to the ADA criterion as HbA_1c_ 5.7% to 6.4% (39 to 47 mmol/mol). We further split the prediabetes group into HbA_1c_ 5.7% to 5.9% (39 to 41 mmol/mol) and HbA_1c_ 6.0% to 6.4% (42 to 47 mmol/mol). We calculated two different indices of insulin sensitivity, reflecting different aspects of insulin sensitivity. The insulin sensitivity index (ISI_0-120_) was calculated as a measure of whole-body insulin sensitivity using fasting and 2hPG and serum insulin concentrations (19). The homeostatic model assessment (HOMA) was used to estimate insulin sensitivity (1/HOMA-insulin resistance) in the fasting state (20), mainly reflecting hepatic insulin sensitivity. HOMA-*β* was calculated as a measure of *β*-cell function (20).

### Assessment of clinical characteristics

At all clinical examinations, anthropometric measures (weight, height, waist circumference) and blood pressure were measured according to standard protocols (21). Information on smoking status and occupation was gathered from questionnaire. During all phases, blood samples were handled according to standardized procedures. Plasma glucose was measured by the glucose oxidase method (22), serum insulin by in-house radioimmunoassays (23), and cholesterol and triglyceride concentrations by automated enzymatic colorimetric methods. Low-density lipoprotein cholesterol was calculated with the Friedewald formula.

### Ethics

The UK National Health Service Health Research Authority London–Harrow Ethics Committee reviewed and approved the study. Written informed consent was obtained from each participant at each examination phase. The study was conducted according to the principles of the Helsinki Declaration. Whitehall II data, protocols, and other metadata are available to *bona fide* researchers for research purposes. Please refer to the Whitehall II data sharing policy at http://www.ucl.ac.uk/whitehallII/data-sharing.

### Statistical analysis

In linear regression models, we studied the association of 5-year changes in glycemic outcomes with concurrent 5-year changes in LPA, MVPA, and TPA (MET-hours/week) adjusting for age, sex, study phase, occupation, and baseline value of PA and the outcome studied. LPA and MVPA were also adjusted for TPA, so the interpretation of the results was that an increase in LPA was at the expense of a decrease in MVPA and *vice versa* (*i.e.*, isotemporal substitution). The following outcomes were studied: FPG, 2hPG, HbA_1c_, HOMA-IS, HOMA-*β*, and ISI_0-120_. Outcomes with a skewed distribution (HOMA-IS, HOMA-*β*, and ISI_0-120_) were log-transformed prior to analysis. Except for HbA_1c_, which was only measured at phases 7 and 9, the same individual could contribute with up to three phases of examinations, which gave rise to two 5-year periods of change in the analysis of glucose-based prediabetes. To account for the likely correlation of repeated measurements within the same participant, we used mixed-effects models with a random intercept. In a sensitivity analysis, we further assessed the mediating effect of 5-year change in body mass index (BMI) on the associations. In the analysis of HbA_1c_ defined prediabetes, we only had data for phases 7 and 9, and therefore, a standard linear model was used for all outcomes. In a sensitivity analysis, we limited the analysis to phases 7 and 9 for the group with prediabetes by the glucose criteria to explore the influence of phase 5 on the results (n = 649).

The associations between baseline PA levels (LPA, MVPA, and TPA) and reversion to normoglycemia after 5 years were studied in age- and sex-adjusted Poisson regression models with follow-up time as offset. LPA and MVPA were additionally adjusted for TPA. We also tested for a modifying effect of prediabetes subgroup on the association between PA levels and the probability of reversion to normoglycemia.

To further study potential heterogeneity in the effect of PA on reversion to normoglycemia, we used recursive partitioning modeling, including age, sex, BMI (normal weight, overweight, obese), prediabetic subgroup, LPA, MVPA, and TPA as explanatory variables. Recursive partitioning analysis is an exploratory method for identifying risk factors and interactions among risk factors that may explain variation in a binary outcome. At each node, the recursive partitioning algorithm identifies the risk factor and split in this factor with the highest discrimination power among all the factor-split combinations at the node. For the development of the present model, the chosen factor-split combination in each node was the one that gave the maximal difference in the probability of reversion to normal glucose tolerance (NGT) between the two resulting subgroups. This procedure was applied recursively until the model was grown to an optimal number of terminal nodes, meaning that further splitting did not improve discrimination between participants. Statistical analyses were performed in R version 3.2.3 and SAS version 9.4. A two-sided 5% level of significance was used.

## Results

### Characteristics of the study population

#### Prediabetes by the glucose criteria

Characteristics of the study participants with prediabetes by the glucose criteria at their first examination are shown in Table 1. The proportion of men was higher among individuals with i-IFG compared with individuals with i-IGT or combined IFG+IGT. People with combined IFG+IGT had in general a worse cardiometabolic risk profile than those with the isolated forms of prediabetes. Mean LPA, MVPA, or TPA levels did not differ between the prediabetic groups at baseline (Table 1).

**Table 1.** **Baseline Characteristics of the Study Population by Prediabetic Criteria**

	**Prediabetes by Glucose Criteria**				**Prediabetes by HbA_1c_ Criterion**		
	**i-IFG**	**i-IGT**	**IFG+IGT**	***P***	**HbA_1c_ 5.7% to 5.9%**	**HbA_1c_ 6.0% to 6.4%**	***P***
Participants	536	305	116		369	88	
Men, %	86.6 (83.4 to 89.3)	75.1 (69.8 to 79.8)*^a^*	78.4 (69.9 to 85.5)*^a^*	<0.001	72.1 (67.2 to 76.6)	70.5 (59.8 to 79.7)	0.761
Age, y	57.2 (6.0)	59.7 (6.4)*^a^*	60.1 (6.5)*^a^*	<0.001	62.2 (6.1)	62.0 (5.7)	0.786
BMI, kg/m^2^	27.2 (3.8)	26.8 (4.2)*^a^*	28.2 (4.4)*^a^*	0.009	27.6 (4.2)	28.4 (4.8)	0.115
Waist circumference, cm	95.7 (10.6)	93.5 (11.8)*^a^*	97.3 (10.6)*^b^*	0.003	96.5 (11)	98.0 (11.3)	0.235
Total cholesterol, mmol/L	5.9 (1.0)	5.9 (1.1)	5.9 (1.0)	0.978	5.8 (1.1)	5.7 (1.0)	0.156
Triglycerides, mmol/L	1.4 (0.9)	1.5 (0.8)	1.7 (1.0)*^a^*	0.004	1.6 (1.1)	1.6 (0.9)	0.626
Systolic BP, mm Hg	126.9 (15.7)	127.8 (17.1)	133.8 (15.8)*^a^*^,^*^b^*	<0.001	129.2 (15.8)	131.6 (19.8)	0.234
Diastolic BP, mm Hg	76.9 (9.8)	76.3 (10.9)	79.5 (10.1)*^a^*^,^*^b^*	0.017	75.5 (9.8)	75.6 (12.3)	0.954
Fasting plasma glucose, mmol/L	5.9 (0.3)	5.1 (0.4)*^a^*	6.1 (0.3)*^a^*^,^*^b^*	<0.001	5.5 (0.6)	5.6 (0.6)	0.149
2hPG, mmol/L	5.8 (1.1)	8.7 (0.8)*^a^*	8.9 (0.9)*^a^*	<0.001	6.7 (1.7)	7.5 (1.8)	0.001
HbA_1c_, %	5.3 (0.3)	5.2 (0.4)*^a^*	5.5 (0.4)*^a^*^,^*^b^*	<0.001	5.8 (0.1)	6.1 (0.1)	<0.001
HbA_1c_, mmol/mol	39.9 (4.0)	38.9 (4.3)*^a^*	42.7 (4.9)*^a^*^,^*^b^*	<0.001	39.6 (0.9)	43.1 (1.2)	<0.001
Fasting serum insulin, pmol/L	8.9 (8.5 to 9.3)	7.8 (7.3 to 8.4)*^a^*	10.6 (9.5 to 11.7)*^a^*^,^*^b^*	<0.001	8.6 (8.0 to 9.3)	9.7 (8.2 to 11.6)	0.171
2-h serum insulin, pmol/L	34.0 (31.8 to 36.4)	73.7 (68.8 to 79.0)*^a^*	75.8 (67.9 to 84.5)*^a^*	<0.001	48.3 (43.1 to 54.1)	55.9 (44.2 to 70.6)	0.254
HOMA-IS	0.42 (0.41 to 0.45)	0.56 (0.52 to 0.6)	0.35 (0.32 to 0.39)	<0.001	0.48 (0.45 to 0.52)	0.39 (0.32 to 0.47)	0.013
HOMA-*β*	73.1 (69.7 to 76.7)	99.6 (93.0 to 106.7)*^a^*	82.3 (74.2 to 91.2)*^a^*^,^*^b^*	<0.001	87 (80.5 to 94.0)	94.4 (78.7 to 113.1)	0.369
ISI_0-120_	35.2 (34.2 to 36.3)	21.4 (20.9 to 21.9)*^a^*	19.8 (19.0 to 20.7)*^a^*^,^*^b^*	<0.001	30.1 (28.6 to 31.7)	26.3 (23.5 to 29.5)	0.089
LPA, MET-h/wk	17.9 (17.0 to 18.9)	17.8 (16.5 to 19.3)	18.2 (15.9 to 20.9)	0.961	17.1 (15.7 to 18.6)	15.7 (13.7 to 18)	0.354
MVPA, MET-h/wk	13.8 (12.6 to 15.2)	13.8 (12.2 to 15.7)	14.8 (12.2 to 18.0)	0.818	12.3 (10.9 to 13.8)	9.6 (7.4 to 12.3)	0.064
TPA, MET-h/wk	34.4 (32.6 to 36.1)	33.4 (30.9 to 36.1)	33.1 (29.1 to 37.6)	0.759	31.1 (28.8 to 33.7)	28.1 (24.4 to 32.3)	0.257
Current smoker, %	8.6 (6.4 to 11.3)	6.6 (4.1 to 9.9)	12.1 (6.8 to 19.4)	0.194	9.8 (6.9 to 13.3)	9.1 (4.0 to 17.1)	0.848
Administrative employment, %	40.7 (36.5 to 45.0)	33.4 (28.2 to 39)	37.1 (28.3 to 46.5)	0.112	30.9 (26.2 to 35.9)	27.3 (18.3 to 37.8)	0.503
Alcohol intake, units/wk	13.4 (12.3 to 14.5)	10.5 (9.3 to 11.8)*^a^*	11.0 (9.1 to 13.3)	0.002	8.0 (7.2 to 9.0)	10.9 (8.2 to 14.4)	0.030
Antihypertensive treatment, %	17.0 (13.9 to 20.4)	23.9 (19.3 to 29.1)*^a^*	30.2 (22.0 to 39.4)*^a^*	0.002	28.7 (24.2 to 33.6)	44.3 (33.7 to 55.3)	0.006
Lipid-lowering treatment, %	5.8 (4.0 to 8.1)	6.6 (4.1 to 9.9)	11.2 (6.1 to 18.4)	0.140	16.0 (12.4 to 20.1)	22.7 (14.5 to 32.9)	0.144

Abbreviation: BP, blood pressure.

Data are means (standard deviation), geometric means (95% CI), or proportions (95% CI). *P* is overall test of difference between groups.

^a^ Versus i-IFG.

^b^ Versus i-IGT.

#### Prediabetes by the HbA_1c_ criterion

Baseline characteristics of individuals with prediabetes by the HbA_1c_ criteria are shown in Table 1. Individuals with higher HbA_1c_ levels had higher mean 2hPG levels and higher alcohol intake than individuals with lower HbA_1c_ levels. The levels of LPA, MVPA, and TPA and most other parameters did not differ between people with lower vs higher HbA_1c_ levels, although there was a tendency for a lower level of MVPA in those with the highest HbA_1c_ levels (Table 1).

### Relationship between 5-year changes in PA and changes in markers of glycemia

#### Prediabetes by the glucose criteria

The associations of 5-year changes in PA with concomitant 5-year changes in glycemia are presented in Table 2. Changes in LPA, MVPA, or TPA were not associated with changes in FPG, 2hPG, or HbA_1c_, but the associations of higher levels of MVPA (at the expense of lower levels of LPA) with reduction in 2hPG levels approaching statistical significance (*P =* 0.060). Also, a 5-year increase of 10 MET-h/wk in MVPA, at the expense of a similar decrease in LPA, was associated with a 3% to 4% improvement in insulin sensitivity and reduction in HOMA-*β* (Table 2). Further adjustment for 5-year changes in BMI did not change the results (Supplemental Table 1). Limiting the analysis to phases 7 and 9 only, the CIs of the point estimates became slightly wider, but the conclusions were similar (Supplemental Table 2). Additionally, a 10 MET-h/wk increase in MVPA at the expense of a decrease in LPA from phase 7 to 9 was significantly associated with a 0.2 mmol/L reduction in the 2hPG level in the sensitivity analysis (Supplemental Table 1).

**Table 2.** **Change in Glucose-Related Outcome (95% CI) by 10 MET Hours per Week Higher Level of LPA, MVPA, or TPA During 5 Years of Follow-Up in Individuals With Prediabetes Diagnosed by the Glucose vs the HbA_1c_ Criteria**

	**LPA**		**MVPA**		**TPA**	
	**Change**	***P***	**Change**	***P***	**Change**	***P***
Prediabetes by glucose criteria (n = 957)						
Fasting plasma glucose, mmol/L	0.00 (–0.04 to 0.05)	0.829	0.00 (–0.05 to 0.04)	0.829	0.00 (–0.02 to 0.02)	0.678
2hPG, mmol/L	0.12 (–0.01 to 0.24)	0.060	−0.12 (–0.24 to 0.01)	0.060	0.00 (–0.05 to 0.06)	0.876
HbA_1c_, % point	0.00 (–0.03 to 0.03)	0.956	0.00 (–0.03 to 0.03)	0.956	0.00 (–0.01 to 0.02)	0.618
HbA_1c_, mmol/mol	0.01 (–0.29 to 0.30)	0.956	−0.01 (–0.3 to 0.29)	0.956	0.03 (–0.10 to 0.17)	0.618
HOMA-IS, % diff	−3.9 (–6.5 to –1.3)	0.004	4.1 (1.3 to 7.0)	0.004	0.9 (–0.3 to 2.2)	0.154
HOMA-*β*, % diff	3.6 (1.1 to 6.2)	0.004	−3.5 (–5.8 to –1.1)	0.004	−0.9 (–2.0 to 0.2)	0.112
ISI_0-120_, % diff	−3.2 (–5.1 to –1.3)	0.0010	3.3 (1.3 to 5.3)	0.001	0.3 (–0.6 to 1.2)	0.512
Prediabetes by HbA_1c_ criterion (n = 457*^a^*)						
Fasting plasma glucose, mmol/L	−0.01 (–0.09 to 0.07)	0.818	0.01 (–0.07 to 0.09)	0.818	0.03 (0.00 to 0.06)	0.078
2hPG, mmol/L	0.15 (–0.16 to 0.46)	0.341	−0.15 (–0.46 to 0.16)	0.341	0.01 (–0.11 to 0.14)	0.835
HbA_1c_, % point	0.03 (–0.01 to 0.06)	0.150	−0.03 (–0.06 to 0.01)	0.150	0.01 (–0.01 to 0.02)	0.500
HbA_1c_, mmol/mol	0.29 (–0.11 to 0.69)	0.150	−0.29 (–0.69 to 0.11)	0.150	0.06 (–0.11 to 0.22)	0.500
HOMA-IS, % diff	−1.3 (–7.7 to 5.4)	0.692	1.4 (–5.2 to 8.3)	0.692	−0.2 (–2.8 to 2.5)	0.884
HOMA-*β*, % diff	−0.9 (–6.7 to 5.1)	0.758	0.9 (–4.9 to 7.1)	0.758	−1.9 (–4.2 to 0.4)	0.110
ISI_0-120_, % diff	−1.0 (–5.9 to 4.2)	0.704	1.0 (–4.0 to 6.2)	0.704	0.6 (–1.5 to 2.6)	0.598

All analyses are adjusted for age, sex, study phase, occupation, and baseline value of PA and the outcome studied. MVPA and LPA are further adjusted for TPA.

^a^ Except for HbA_1c_, only the subset fasting ≥8 hours at both baseline and follow-up were used in the analyses (n = 250).

#### Prediabetes by the HbA_1c_ criterion

Among individuals with prediabetes by HbA_1c_, 5-year changes in LPA, MVPA, or TPA were not associated with reductions in HbA_1c_ or with changes in any of the glucose-related markers (Table 2). Adjustment for 5-year changes in BMI did not change the results substantially. However, an increase in PA was associated with 4 mmol/L higher fasting plasma glucose concentration in the BMI-adjusted analysis (Supplemental Table 1).

### Relationship of baseline PA with 5-year reversion to normoglycemia

#### Prediabetes by the glucose criteria

During the follow-up period, 405 (42%) individuals reverted to NGT. Mean [95% confidence interval (CI)] 5-year reversion probabilities to NGT status were 31.9% (95% CI, 28.8 to 35.3) in individuals with i-IFG, 31.0% (95% CI, 26.7 to 35.7) in i-IGT and 18.5% (95% CI, 13.5 to 25.2) in combined IFG+IGT. We did not find a modifying effect of prediabetic subgroup on the association between PA and reversion to NGT (*P* ≥ 0.554). Also, in the entire prediabetic population LPA, MVPA, or TPA at baseline were not significantly associated with the probability of reversion to NGT (*P* ≥ 0.085 for all).

**Figure 1.**
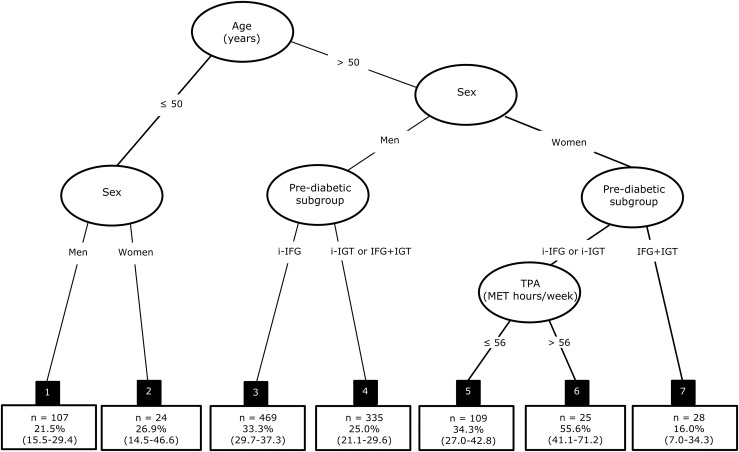
Survival tree for reversion to NGT (prediabetes by glucose criteria). The black boxes 1 to 7 are the seven terminal nodes of the tree, each with the number (n) of 5-year periods of change and their mean 5-year probability of reversion to NGT with 95% CI.

Using recursive partitioning, we identified subgroups in which TPA was associated with reversion to NGT (Fig. 1, terminal nodes). The most significant predictor of reversion to NGT was age, and the optimal split was at 50 years of age (Fig. 1, top). Among individuals below 50 years of age, sex was also associated with reversion to NGT (*P* = 0.024). Here, the mean 5-year probability of reversion to NGT was slightly lower among men (21.5%, node 1) than among their female counterparts (26.9%, node 2). For individuals aged 50 years or above, sex was also significantly associated with reversion to NGT (*P* = 0.035). In addition, among men, prediabetic subgroup was associated with reversion to NGT (*P* = 0.031). Here men with i-IFG had a higher 5-year probability of reversion to NGT (33.3%, node 3) than those with i-IGT or combined IFG+IGT (25.0%, node 4). Among older women, prediabetic subgroup was also associated with reversion to NGT with lower reversion probability in those with IFG+IGT than the groups with i-IFG or i-IGT (*P* = 0.021). Additionally, among older women with i-IFG or i-IGT the amount of TPA was associated with the probability of reversion to NGT (*P* = 0.032). The optimal split was at 56 MET-hours. Those with a weekly TPA level of ≤56 MET-hours had a mean 5-year probability of reversion to NGT of 34.3% (node 5). In contrast, 55.6% of those with a weekly TPA level of >56 MET-hours reverted to NGT (node 6). Among older women with IFG+IGT, the 5-year probability of reversion to NGT was only 16.0% and this was not modified by baseline PA level (node 7).

By further studying the subgroups resulting from the recursive partitioning analysis (nodes 1 to 7), we found that the groups differed by other baseline characteristics than those included in the model (Table 3). None of the women ≥50 years with i-IFG or i-IGT who reported a high amount of TPA was a smoker or used lipid-lowering treatment at baseline (node 6). Also, it was seen that older women with IFG+IGT (node 7) had lower insulin sensitivity at baseline compared with those with i-IFG or i-IGT (node 6, *P* < 0.001). Among men ≥50 years, those with i-IFG (node 3) had a higher level of ISI_0-120_ and lower level of HOMA-*β* than those with i-IGT or IFG+IGT (node 4, *P* < 0.001 for both).

**Table 3.** **Baseline Characteristics of the Study Population by Terminal Node of the Survival Tree**

	**Node 1**	**Node 2**	**Node 3**	**Node 4**	**Node 5**	**Node 6**	**Node 7**	***P***
Number of 5-y periods of change	107	24	469	335	109	25	28	
Men, %	100	0	100	100	0	0	0	<0.001
Age, y	48.7 (1.4)	49.1 (1.5)	59.2 (5.2)	61.2 (5.8)	58.6 (4.7)	61.7 (6.0)	60.9 (6.3)	<0.001
BMI, kg/m^2^	27.6 (4.0)	29.3 (5.1)	27.1 (3.8)	27.2 (3.9)	27.3 (5.0)	26.5 (4.5)	29.1 (6.1)	0.042
HOMA-IS	0.40 (0.36 to 0.45)	0.41 (0.33 to 0.51)	0.43 (0.41 to 0.45)	0.47 (0.44 to 0.51)	0.51 (0.45 to 0.58)	0.55 (0.41 to 0.74)	0.34 (0.27 to 0.41)	<0.001
HOMA-*β*	87.8 (77.9 to 99.0)	85.4 (69.8 to 104.6)	71.5 (68.0 to 75.2)	90.6 (85.0 to 96.5)	91.4 (80.7 to 103.6)	83.0 (63.5 to 108.5)	87.1 (71.6 to 105.8)	<0.001
ISI_0-120_	30.9 (28.3 to 33.9)	27.1 (23.4 to 31.4)	35.0 (34.0 to 36.1)	20.9 (20.4 to 21.3)	26.8 (25.3 to 28.4)	25.7 (22.6 to 29.2)	19.2 (17.8 to 20.8)	<0.001
Current smoker, %	16.8 (10.3 to 25.3)	4.2 (0.1 to 21.1)	7.2 (5.1 to 10.0)	5.7 (3.4 to 8.7)	11.0 (5.8 to 18.4)	0	14.3 (4.0 to 32.7)	0.004
Administrative employment, %	36.4 (27.4 to 46.3)	4.2 (0.1 to 21.1)	45.4 (40.8 to 50.0)	41.5 (36.2 to 47.0)	19.3 (12.3 to 27.9)	16.0 (4.5 to 36.1)	17.9 (6.1 to 36.9)	<0.001
LPA, MET-h/wk	15.6 (13.7 to 17.9)	19.1 (14.9 to 24.6)	17.8 (16.9 to 18.9)	17.3 (16.0 to 18.7)	19.4 (17.7 to 21.4)	39.1 (34.2 to 44.5)	19.9 (15.1 to 26.3)	<0.001
MVPA, MET-h/wk	9.3 (7.3 to 12.0)	11.2 (6.6 to 19.2)	15.1 (13.7 to 16.6)	16.3 (14.7 to 18.1)	6.2 (5.0 to 7.7)	29.0 (23.1 to 36.4)	11.8 (7.4 to 18.7)	<0.001
TPA, MET-h/wk	26.9 (23.4 to 30.9)	31.3 (24.2 to 40.4)	35.9 (34 to 37.9)	35.8 (33.4 to 38.3)	25.8 (23.3 to 28.6)	70.9 (64.7 to 77.6)	28.5 (20.9 to 38.9)	<0.001
Antihypertensive treatment, %	6.5 (2.7 to 13.0)	16.7 (4.7 to 37.4)	19.2 (15.7 to 23.1)	30.1 (25.3 to 35.4)	18.3 (11.6 to 26.9)	20.0 (6.8 to 40.7)	25.0 (10.7 to 44.9)	<0.001
Lipid lowering treatment, %	2.8 (0.6 to 8.0)	4.2 (0.1 to 21.1)	7.5 (5.3 to 10.2)	10.4 (7.4 to 14.2)	4.6 (1.5 to 10.4)	0	3.6 (0.1 to 18.3)	0.022

Data are means (standard deviation), geometric means (95% CI), or proportions (95% CI). *P* is overall test of difference between nodes.

#### Prediabetes by the HbA_1c_ criterion

During 5 years of follow-up, only 20 (4.4%) individuals with HbA_1c_-defined prediabetes reverted to normoglycemia (HbA_1c_ < 5.7%/39 mmol/mol). Five-year reversion probabilities to normoglycemia were 4.7% (3.0 to 7.4) in individuals with HbA_1c_ 5.7% to 5.9% (39 to 41 mmol/mol) and 2.2% (0.6 to 8.6) in individuals with HbA_1c_ 6.0% to 6.4% (42 to 47 mmol/mol). We did not find a modifying effect of HbA_1c_ subgroup on the association between baseline PA and reversion to normoglycemia (*P* ≥ 0.107), and neither PA, age, sex, BMI, nor the level of HbA_1c_ was associated with reversion to normoglycemia (*P* ≥ 0.255 for all). Hence, a recursive partitioning model could not be made for this group.

## Discussion

It is well documented that lifestyle intervention including high levels of PA can delay or even prevent the development of type 2 diabetes in individuals with IGT (4–6), but the evidence is less clear in individuals with prediabetes identified by FPG or HbA_1c_. We found that an increase in MVPA over time at the expense of a decrease in LPA was associated with subtle improvements in glycemic markers in individuals with prediabetes defined by the glucose criteria. PA was not a strong determinant for 5-year reversion to normoglycemia in the entire prediabetic population, but TPA was associated with 5-year reversion to NGT in women with i-IFG or i-IGT aged 50 years or above. Reversion to normoglycemia was rare among people with prediabetes based on the HbA_1c_ criterion, and PA was not associated with improvements in glycemic markers in this group.

We hypothesized that individuals with i-IFG would have a smaller effect of PA levels on improvement of glycemia than individuals with i-IGT or IFG+IGT, but this could not be confirmed in the current study. Yet, the fact that an increase in MVPA was associated with improvements in 2hPG and insulin sensitivity, but not with reductions in FPG levels, support the notion that fasting hyperglycemia is not modifiable by lifestyle factors to the same extent as hyperglycemia after an OGTT (10, 24). A previous longitudinal, observational, study found that physical inactivity is not associated with progression to type 2 diabetes in individuals with i-IFG (8). In support of these findings, another study revealed that individuals with i-IFG have the same levels of objectively measured daily PA and cardiorespiratory fitness as individuals with NGT (9). In the current study, the levels of self-reported LPA, VPA, and TPA did not differ between the different prediabetic groups. However, we found that individuals with i-IFG—and, particularly, men ≥50 years—had better whole-body insulin sensitivity (ISI_0-120_) at baseline and a higher probability of reversion to NGT than those with i-IGT or IFG+IGT. These findings could suggest that differences in PA and other lifestyle-related factors were present in the years before the baseline examination.

Of the 457 individuals classified as having prediabetes by the HbA_1c_ criterion, only 20 reverted to normoglycemia during 5 years of follow-up, and we did not find any determinants of reversion to normoglycemia in this group. This finding underscores that individuals identified by HbA_1c_ represent a different group than those identified by the FPG or 2hPG criteria (25–27). A *post hoc* analysis of the DPP supports this notion. It was found that lifestyle intervention was not superior to metformin on diabetes risk reduction when HbA_1c_ was used as the diagnostic tool instead of glucose (28). Our findings also emphasize that HbA_1c_ is a much more stable tool for identifying prediabetes than fasting and 2-hour glucose, which have high day-to-day variation (29) and thereby a higher probability of misclassification and reversion to NGT. Surrogate markers of insulin sensitivity and *β*-cell function based on fasting and post-OGTT glucose and insulin levels acutely respond to subtle changes in PA and diet (30). In contrast, HbA_1c_ reflects the average glycemic level over the last 8 to 12 weeks and is thereby less responsive to daily behavioral changes (31). The mean levels of FPG and 2hPG as well as insulin sensitivity and beta cell function were in the normal range in participants classified as having prediabetes by HbA_1c_. Hence, the potential for improvement was also smaller in this group than in those identified by the OGTT.

The general lack of association between PA and reduction in HbA_1c_ is supported by previous research, where no associations of PA energy expenditure or cardiorespiratory fitness with HbA_1c_ were found in a high-risk population after adjustment for age, sex and obesity degree (9). However, a small intervention study in 21 overweight and obese individuals with prediabetes identified by HbA_1c_ showed that 16 weeks of supervised high-intensity interval or continuous moderate-intensity training combined with resistance training resulted in a mean reduction in HbA_1c_ of 0.5% (∼5 mmol/mol) as well as improvements in both insulin sensitivity and beta cell function assessed by the HOMA model (32). Higher obesity degree and higher baseline HbA_1c_ levels of the study population together with the long-term, supervised, high-intensity intervention is likely to explain the beneficial effects observed in this small study as compared with our observational study.

An interesting finding from our study was that TPA was particularly important for older women with i-IFG or i-IGT in terms of normalizing their blood glucose levels. A number of studies examining the effect of different lifestyle interventions on diabetes prevention in individuals with prediabetes have studied whether the effect of the various interventions differ across sex and age in subgroup analyses (33). Most intervention studies found no sex differences in the effect of lifestyle interventions on diabetes prevention or changes in glycemic parameters in individuals with prediabetes (33, 34), potentially because they were underpowered to look at those interactions or because higher order interactions with other parameters (*e.g.*, age, obesity degree and prediabetic subgroup) were not examined. However, in the DPP it was found that among individuals with combined IFG+IGT men tended to be more likely than women to revert to NGT (16). Also the DPP study found that men were more likely to revert from combined IFG+IGT to i-IFG, whereas women were more likely to revert from combined IFG+IGT to i-IGT (16). This observation emphasizes differences in the sex distribution across the prediabetic subgroups shown in this study as well as in many other studies (35–38). Our finding that age was an important determinant for reversion to normoglycemia is also supported by results from the DPP study showing that younger individuals were more likely to revert from prediabetes to NGT than older individuals (16). In terms of preventing diabetes development (in contrast to reversion to NGT), the Finnish Diabetes Prevention Study and the DPP found that the effect of lifestyle intervention was greatest in older age groups (4, 34), which was in alignment with our finding where TPA was mainly predictive of reversion to NGT in women aged 50 years or above. A similar conclusion was made from a meta-analysis of twelve intervention studies (39). The cut-point for TPA of 56 MET-h/wk was derived from the statistical model as the optimal cut-point for discriminating between study participants with different probabilities of reversion to NGT. An amount of PA of 56 MET-h/wk (∼8 MET-h/d) can be achieved by, for example, 1 hour of brisk walking/light bicycling and 30 minutes of running/jogging each day.

Strengths of this study were the long follow-up time and the detailed clinical data, including OGTTs, collected on a large number of individuals. Also, the availability of concomitant measurements of glycemic markers and PA facilitated modeling of temporal changes in PA patterns and use of the isotemporal substitution model (40). This model has become more common in recent years, and has a clear advantage because of the easier interpretation of substituting one type or intensity of PA with another. Furthermore, the use of recursive partitioning as a statistical method enabled us to identify subgroups of prediabetic individuals who may particularly benefit from increasing their PA to normalize their blood glucose levels. This finding would not have been revealed by simple regression analysis, as we found no overall association between PA and reversion to NGT in the entire prediabetic population. A limitation of using recursive partitioning is that some of the identified subgroups can be relatively small. However, because this analysis was not focused on developing a prediction model for reversion to NGT but rather on a deeper understanding of the associations between PA and improvement in glycemia, we did not want to include a minimum group size in the analysis. Another important issue to mention is the number of tests performed in the analyses of associations between changes in PA and glycemia markers. We did not adjust for multiple testing in the results, because the outcomes were predefined and highly correlated. However, even with adjustment for multiple testing (41), the observed associations remained significant.

The Whitehall II study is an occupational cohort consisting predominantly of white-collar workers, and therefore, a certain degree of healthy worker effect may be present in our study. Accordingly, our population may be more homogeneous in terms of health status and PA compared with the general population, which may limit the possibility to detect meaningful associations. Detailed information on habitual PA was assessed using a 20-item questionnaire, allowing the quantification of a broad range of activities, which were translated into intensities using reference MET values (18). Although the questionnaire gives detailed information about PA behavior, self-report measures of PA tend to overestimate PA levels as compared with objectively measured PA (42). More importantly, misreporting of PA seems to differ across populations and subgroups of participants. A study found that a 24-hour PA recall underestimated MVPA for younger normal weight individuals, but overestimated MVPA for older, more obese individuals (43). This suggests that the absolute levels of MVPA may be overestimated in this rather homogeneous group of older prediabetic individuals from the Whitehall II study, but with no indication of differential misreporting across the population. Accordingly, the reported associations are likely unbiased from misreporting of PA.

In conclusion, among individuals with prediabetes defined by the glucose criteria, substituting LPA with MVPA was associated with improvements in 2hPG and insulin sensitivity. We also showed that a high level of TPA was particularly important for reversion to normoglycemia among women aged ≥50 years with i-IFG or i-IGT. Individuals identified as having prediabetes by HbA_1c_ had a low reversion rate to normoglycemia, and their changes in glycemia were not associated with PA. These findings highlight that heterogeneity in prediabetes exists and that one-size-fits-all strategies for diabetes prevention may not be feasible. Our results also question whether results from large randomized diabetes prevention trials in individuals with IGT (4–6) can be applied to individuals identified with prediabetes by HbA_1c_. Indeed, more evidence is needed regarding early prevention of type 2 diabetes in individuals identified with prediabetes by the HbA_1c_ method (44).
