# Supplementary material for: Physical Activity and Improvement of Glycemia in Prediabetes by Different Diagnostic Criteria
Source: J Clin Endocrinol Metab. 2017 Jul 26;102(10):3712–21. doi: 10.1210/jc.2017-00990 (PMC5630255; doi:10.1210/jc.2017-00990)
Supplement: Supplementary file 1 [file jc.2017-00990.st1.pdf]

Table S1: Change in glucose-related outcome (95% CI) by 10 MET hours/week higher level of light-intensity physical activity (LPA), moderate-to-vigorous-intensity physical activity (MVPA) or total physical activity (TPA) during 5-years of follow-up in individuals with pre-diabetes diagnosed by the glucose vs. the HbA_{1c} criteria with further adjustment for 5-year change in BMI.

	**LPA**		**MVPA**		**TPA**	
	**Change**	***P***	**Change**	***P***	**Change**	***P***
Pre-diabetes by glucose criteria (n=957)						
Fasting plasma glucose (mmol/l)	0.00 (-0.04;0.05)	0.955	0.00 (-0.05;0.04)	0.955	0.00 (-0.02;0.02)	0.793
2-hour plasma glucose (mmol/l)	0.11 (-0.01;0.24)	0.071	-0.11 (-0.24;0.01)	0.071	0.01 (-0.05;0.07)	0.783
HbA_{1c} (%-point)	-0.01 (-0.03;0.02)	0.639	0.01 (-0.02;0.03)	0.639	0.01 (-0.01;0.02)	0.374
HbA_{1c} (mmol/mol)	-0.07 (-0.36;0.22)	0.639	0.07 (-0.22;0.36)	0.639	0.06 (-0.07;0.19)	0.374
HOMA-IS (% difference)	-2.8 (-5.3;-0.3)	0.029	2.9 (0.3;5.6)	0.029	0.4 (-0.7;1.6)	0.470
HOMA-β (% difference)	2.7 (0.3;5.2)	0.026	-2.6 (-4.9;-0.3)	0.026	-0.5 (-1.6;0.5)	0.327
$ISI_{0\text{-}120}$ (% difference)	-2.6 (-4.4;-0.7)	0.007	2.6 (0.7;4.6)	0.007	0.1 (-0.7;1.0)	0.756
Pre-diabetes by HbA_{1c} criterion (n=457)*						
Fasting plasma glucose (mmol/l)	-0.01 (-0.09;0.06)	0.717	0.01 (-0.06;0.09)	0.717	0.04 (0.00;0.07)	0.031
2-hour plasma glucose (mmol/l)	0.14 (-0.17;0.45)	0.376	-0.14 (-0.45;0.17)	0.376	0.02 (-0.11;0.15)	0.756
HbA_{1c} (%-point)	0.02 (-0.01;0.06)	0.183	-0.02 (-0.06;0.01)	0.183	0.01 (-0.01;0.02)	0.322
HbA_{1c} (mmol/mol)	0.27 (-0.12;0.66)	0.183	-0.27 (-0.66;0.12)	0.183	0.08 (-0.08;0.24)	0.322
HOMA-IS (% difference)	0.1 (-5.8;6.3)	0.977	-0.1 (-5.9;6.1)	0.977	-1.1 (-3.5;1.3)	0.356
HOMA-β (% difference)	-1.6 (-7.1;4.3)	0.595	1.6 (-4.1;7.6)	0.595	-1.5 (-3.7;0.8)	0.209
$ISI_{0\text{-}120}$ (% difference)	-0.5 (-5.4;4.6)	0.836	0.5 (-4.4;5.7)	0.836	0.2 (-1.8;2.3)	0.826

All analyses are adjusted for age, sex, study phase, occupation, 5-year change in BMI and baseline value of physical activity and the outcome studied. MVPA and LPA are further adjusted for TPA.

Table S2: Change in glucose-related outcome (95% CI) by 10 MET hours/week higher level of light-intensity physical activity (LPA), moderate-to-vigorous-intensity physical activity (MVPA) or total physical activity (TPA) during 5-years of follow-up in individuals with pre-diabetes diagnosed by the glucose criteria using phase 7 and 9 only.

	**LPA**		**MVPA**		**TPA**	
	**Change**	***P***	**Change**	***P***	**Change**	***P***
Pre-diabetes by glucose criteria (n=957)						
Fasting plasma glucose (mmol/l)	0.02 (-0.02;0.07)	0.321	-0.02 (-0.07;0.02)	0.321	0.01 (-0.04;0.05)	0.794
2-hour plasma glucose (mmol/l)	0.19 (0.05;0.32)	0.009	-0.19 (-0.32;-0.05)	0.009	-0.02 (-0.15;0.10)	0.737
HbA_{1c} (%-point)	0.00 (-0.03;0.03)	0.956	0.00 (-0.03;0.03)	0.956	0.01 (-0.01;0.04)	0.374
HbA_{1c} (mmol/mol)	0.01 (-0.29;0.30)	0.956	-0.01 (-0.30;0.29)	0.956	0.12 (-0.14;0.38)	0.374
HOMA-IS (% difference)	-5.2 (-8.2;-2.0)	0.002	5.4 (2.0;9.0)	0.002	1.9 (-1.1;5.0)	0.207
HOMA-β (% difference)	3.7 (0.7;6.8)	0.016	-3.6 (-6.4;-0.7)	0.016	-2.5 (-5.0;0.2)	0.067
ISI_{0-120} (% difference)	-3.6 (-5.7;-1.4)	0.002	3.7 (1.4;6.1)	0.002	1.1 (-1.0;3.2)	0.300

All analyses are adjusted for age, sex, study phase, occupation and baseline value of physical activity and the outcome studied. MVPA and LPA are further adjusted for TPA.
